# Fatal gastrointestinal bleeding in a case report of Coat’s plus syndrome^⋆^

**DOI:** 10.1016/j.ijscr.2019.12.005

**Published:** 2019-12-16

**Authors:** Mohammed Jeraq, Valerie Armstrong, Grigoriy Klimovich, Krishnamurti Amrit Rao, Patricia Byers

**Affiliations:** Dewitt Daughtry Family Department of Surgery, Division of Trauma, Burns, and Critical Care, Jackson Memorial Hospital, University of Miami Miller School of Medicine, Miami, FL, United States

**Keywords:** Coat’s, Coat’s syndrome, Gastrointestinal bleeding, Lower gastrointestinal bleed, Upper gastrointestinal bleed, Case report

## Abstract

**Introduction:**

Coat’s plus syndrome is an extremely rare genetic syndrome that leads to a variety of symptoms. We are reporting a case of Coat's plus syndrome that had persistent GI bleeding and review of current literature.

**Presentation of case:**

The patient is a female in her 40 s with a history of coat's disease and end stage renal failure on dialysis. The etiology of renal failure was not discovered, and the patient was being worked up for a kidney transplant. The patient required admission after deterioration of nutritional status with a BMI of 14.3. During admission the patient initially had intermittent GI bleeding requiring weekly blood transfusions. On work up of the GI bleed, no etiology was identified either. As a result persistent negative GI bleed work up, we pursued alternative diagnoses. The history of Coat’s disease prompted us to work up the patient for Coat's plus syndrome. A genetic test confirmed the presence of CTC-1 gene mutation, which results in Coat's plus syndrome. With no treatment available as of yet, the patient continued to deteriorate into multi-organ failure.

**Discussion:**

We present an example of GI bleeding in Coat's plus syndrome, only identified thru genetic testing, that is very rare and complex in nature. Despite numerous workups, no specific etiology was identified for the GI bleeding.

**Conclusion:**

Previous reports have not investigated cause of GI bleeding, since it is extremely rare in the literature. Further investigation is warranted to understand cause and effects of GI bleeding in this rare genetic disease.

## Highlights

• Coat’s plus syndrome is a rare genetic condition.
• Manifestations of this syndrome include ophthalmologic symptoms along with systemic manifestations such as gastrointestinal bleeding.
• This case report demonstrates significant morbidity and ultimately mortality associated with this condition.
• Further research is needed in this area to treat patients with GI bleeding in Coat’s plus syndrome.

## 1 Introduction

Coat’s plus syndrome is a rare condition with only a handful of cases reported worldwide. This condition results from a genetic mutation in the CTC1 gene that alters telomere function and possibly even telomere structure. This leads to multiple symptoms, including gastrointestinal (GI) bleeding. We are reporting our experience with this rare condition in a female in her 40 s who was experiencing severe malnutrition with subsequent persistent GI bleeding. The work done for this case report is in line with SCARE criteria [8].

## 2 Case description

A female in her 40 s was referred to our surgical nutrition clinic for management of her nutritional status by the transplant nephrologist team. Initially, the patient was being assessed for a kidney transplant. However, her nutritional status had continued to deteriorate, with her BMI decreasing from 16.8 to 14.3 over the course of one year. She had a past medical history significant for Coat’s disease and end-stage renal disease. The patient has been managed for her Coat’s disease with eye enucleation 16 years prior to presentation. Her kidney failure started while the patient was in her 30 s. All laboratory work-up, including for autoimmune diseases and vasculitis were negative. A kidney biopsy was also inconclusive. The patient also had a history of GI bleed several years prior and was diagnosed with gastrointestinal vascular ectasia (GAVE) syndrome necessitating blood transfusion on a monthly basis.

The patient returned to clinic for follow up in 2 months. At this point she was also having shortness of breath at rest, requiring being on CPAP during the night. She was admitted to the hospital from clinic to investigate the cause of her malnutrition and etiology of her shortness of breath. Total parenteral nutrition was initiated immediately to improve her nutritional status acutely. Our team proceeded with working up the patient for her current condition.

We repeated the rheumatologic work up for the patient to assess the presence of any autoimmune disease or vasculitis. All the laboratory panel results were negative. The patient continued to have shortness of breath, requiring being on BIPAP. CT imaging of the chest and an echocardiogram did not reveal cardiac dysfunction that would lead to the dyspnea the patient was experiencing at rest. At this point, it was determined that the patient’s significant generalized weakness and muscle weakness resulted in a poor respiratory effort. Due to poor oral intake while the patient was on a BIPAP machine, a percutaneous gastrojejunosostomy tube was inserted to increase the patient’s enteral nutrition.

**Fig. 1:**
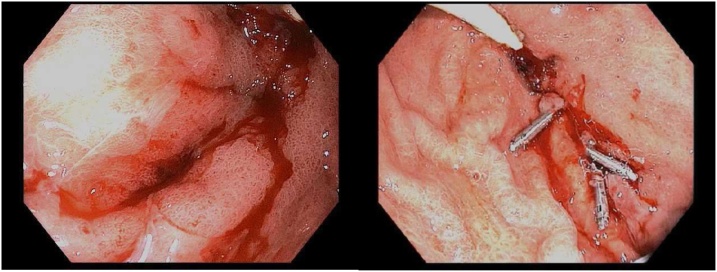
(a) Gastric body ulcer oozing with contact bleeding. (b) Gastric antrum ulcer after clips placed.

In regard to the patient’s GI bleeding, she had already given a history of GAVE syndrome requiring blood transfusions. On admission, the patient was anemic and required blood transfusion on a weekly basis approximately. However, she started to have more frequent GI bleeds, as evidenced by the increasing melena and drop in hemoglobin levels. We started our work-up with an upper GI endoscopy and lower GI endoscopy, which did not reveal any significant pathology (Fig. 1A/B). The patient at this point was on proton-pump inhibitors and receiving blood transfusions as necessary.

**Fig. 2:**
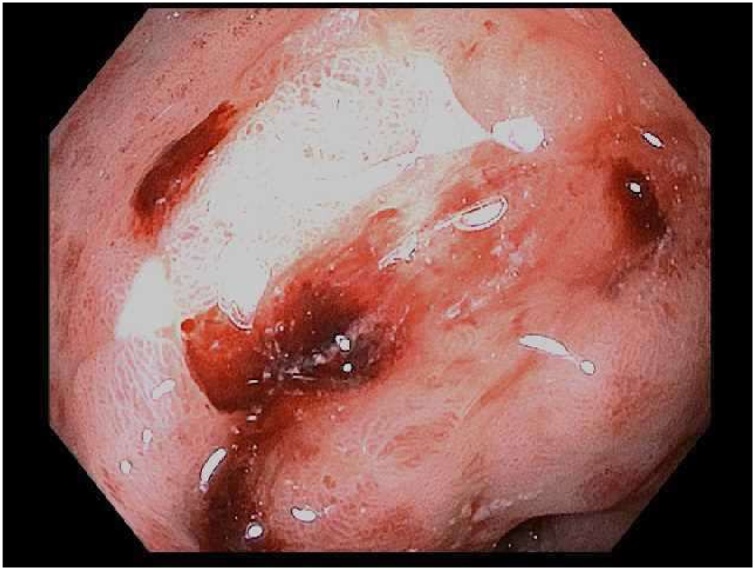
Multiple small angioectasis (hx of GAVE) with bleeding in the pre-pyloric region of stomach. 3 clips placed.

**Fig. 3:**
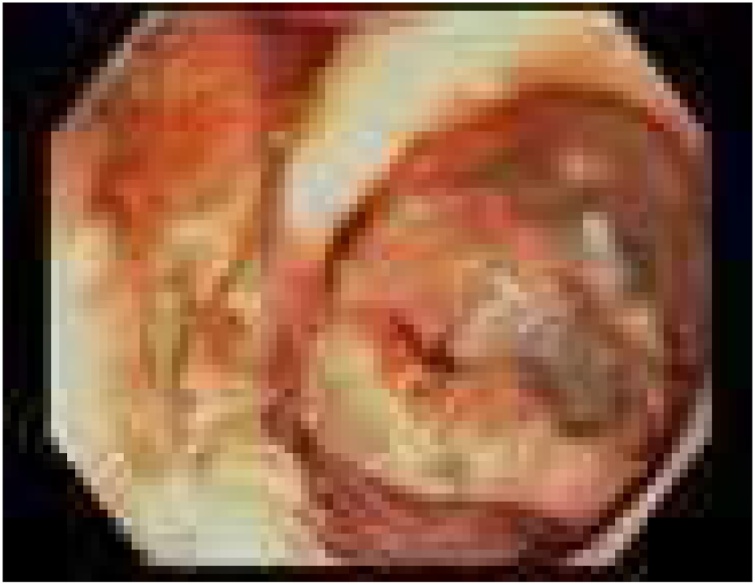
Pre-pyloric ulcer with active bleeding in antrum, likely cause of GIB.

The patient continued to have intermittent GI bleeds that became more frequent and severe with time. Another upper and lower GI endoscopy was performed that showed GAVE (Fig. 2). We proceeded with tagged RBC scan to further delineate the source of the bleeding. The scan, however, did not show a source of bleed. A highly selective celiac and mesenteric artery angiography was also performed, which again did not show any contrast extravasation. Finally, we assessed the patient’s GI bleed with capsule endoscopy, which revealed mucosal blood oozing in the proximal small bowel (Fig. 3).

**Fig. 4:**
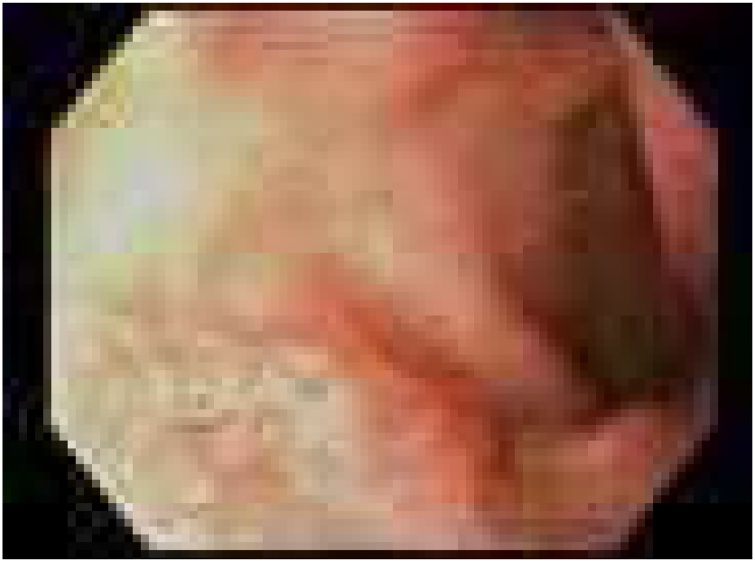
Duodenal bulb bleeding despite OCP, protonix, octreotide, and estrogen.

With all the investigations of a GI bleed in this patient resulting as negative, we evaluated other potential causes. As the patient had a history of coat’s disease, we performed a literature search for a systemic manifestation of Coat’s disease and Coat’s plus syndrome. A few case reports revealed patients who had Coat’s disease and abnormal GI bleeding. Subsequently, we tested our patient for a CTC-1 gene mutation, which came back positive. After a completion of a final upper GI endoscopy (Fig. 4) the patient was started on ortho-cyclen (OCP) and continued protonix, octreotide, and estrogen therapy, as other case reports have achieved some success with these treatments. The patient failed to respond to these management options. Eventually, the patient died secondary to multi-organ failure and sepsis.

## 3 Discussion

Coats plus syndrome is an autosomal recessive disorder caused by mutations in a telomere gene. This complex is crucial for telomere replication [1]. Coats plus patients display abnormally shortened telomeres, suggesting that telomere dysfunction plays a crucial role in pathogenesis [1,2].

Another similar entity is leukoencephalopathy, brain calcifications, and cysts (LCC), which was first described by Labrune et al. in 1996 that characterizes extensive brain calfications, leukodystrophy, and formation of parenchymal cysts [3].

Despite literature describing differences between Coats plus and LCC, they have been collectively referred to as cerebroretinal microangiopathy with calcifications and cysts (CRMCC), and it has previously been proposed that they may share a common genetic mutation [4]. The clinical phenotype of CRMCC is wide and variable, and affected patients may present to specialists in various fields, such as neonatology, ophthalmology, gastroenterology and neurology [4]. However, researchers have suggested that these two groups may be distinguished according to the presence or absence of extra-neurological features, such as individuals without neurological features or skeletal abnormalities or gastrointestinal manifestations [5].

Coats plus syndrome has additional non-neurological features which distinguish it from (LCC), including retinal telangiectasia and exudates, increased incidence of osteopenia and recurrent fractures, and a risk of gastrointestinal bleeding and portal hypertension [5]. The GI bleeding and underlying cirrhosis is caused by vascular ectasia development in the stomach, small intestines and liver [5]. Briggs et al. found two out of eight patients with Coats disease (25 %) who had GI bleeding. Our patient demonstrated GI bleeding with positive CTC1 mutation consistent with Coats plus. A case series by Linnankivi et al. also confirmed the finding of GI bleeding. In that particular case series, the researchers found severe and recurrent GI bleeding and GI vasculature abnormalities, such as thick walled or dilated vessels [6]. In addition, two individuals from that study developed hepatic insufficiency and esophageal varices [5,6]. Another study Van Effenterre et al. reported similar features of GI bleeding, with no treatment as well [7].

However, currently there are no treatment guidelines for gastrointestinal bleeding in Coats plus syndrome. Prior studies did not reveal any options for treatment. In our patient, embolization of bleeding source did not fully resolve the bleeding. Subsequently, the patient died from multi-organ failure secondary to the GI bleeding.

Further research is needed in this area to treat patients with GI bleeding in Coats plus syndrome.

## Sources of funding

No funding.

## Ethical approval

Ethical approval has been exempted by our institution.

## Consent

Consent was not obtained, patient is deceased and next of kin could not be located.

The head of our medical team has taken responsibility that exhaustive attempts have been made to contact the family and that the paper has been sufficiently anonymised not to cause harm to the patient or their family. A copy of this document is available for review by the Editor-in-Chief of this journal on request.

## Author contributions

Mohammed Jeraq: literature search, case report design, gathering history, data interpretation.

Valerie Armstrong: literature search, gathering history, data interpretation, editing.

Grigoriy Klimovich: literature search, cases report design, gathering history, data interpretation.

Krishnamurti Amrit Rao: literature search, conclusion.

Patricia Byers: literature search, case report design, gathering history, data interpretation.

## Registration of research studies

No study was done.

## Guarantor

Mohammed Jeraq.

## Provenance and peer review

Not commissioned, externally peer-reviewed.

## Declaration of Competing Interest

No conflicts of interest.
